# The effects of valproic acid and levetiracetam treatments on kidney functions in children with epilepsy

**DOI:** 10.12669/pjms.41.4.10385

**Published:** 2025-04

**Authors:** Nesrin Tas, Ozge Balci, Mehmet Senes, Sukran Bicakci, Arzu Yılmaz

**Affiliations:** 1 Nesrin Tas, MD, Department of Pediatric Nephrology, University of Health Sciences Ankara Training, Research Hospital, Ankara, Turkey; 2 Ozge Balcı, MD, Department of General Pediatrics, University of Health Sciences Ankara Training, Research Hospital, Ankara, Turkey; 3 Mehmet Senes, MD, Department of Clinical Biochemistry, University of Health Sciences Ankara Training, Research Hospital, Ankara, Turkey; 4 Sukran Bicakci, MD, Department of Clinical Biochemistry, University of Health Sciences Ankara Training, Research Hospital, Ankara, Turkey; 5 Arzu Yilmaz, MD, Department of Pediatric Neurology, University of Health Sciences Ankara Training, Research Hospital, Ankara, Turkey

**Keywords:** Adverse effects, Antiepileptic drugs, Fanconi syndrome, Hyperfiltration, Kidney, Levetiracetam, Valproic acid

## Abstract

**Objectives:**

Clinical and subclinical evidence of kidney dysfunction has been reported with the use of some anti-epileptic drugs (AEDs) in children. This study aimed to evaluate renal tubular and glomerular functions using novel biomarkers N-acetyl-β-d-glycosaminidase (NAG), Kidney Injury Molecule-1(KIM-1) and 24-hour measured creatinine clearance (mCl_cr_) in patients taking valproic acid (VPA) and levetiracetam (LEV).

**Methods:**

Fifty-one epileptic patients were included in this prospective case-control study between January 2023 and January 2024 in The Ankara Training and Research Hospital. Exclusions were made for individuals on multiple AEDs, those with incomplete data, bedridden patients, concurrent conditions (hypertension, obesity), or those using steroids or other medications. Participants had been on either VPA or LEV for at least six months. Sixteen healthy age- and sex-matched children served as the control group. Blood samples and 24-hour urine NAG, KIM-1, and mClcr measurements were collected.

**Results:**

The VPA, LEV, and control group comprised 30, 21, and 16 patients, respectively. The VPA group had higher urinary pH (6.1±0.7) and mCl_cr_ levels (173.4±48.0) than LEV (5.6±0.5, 149.7±39.1) and control (5.6±0.6, 130.6±30.6) groups. No significant difference was observed in urinary pH and mCl_cr_ levels between the LEV and control groups *(p>0.05)*. No statistically significant differences were observed among the VPA, LEV, and control groups regarding 24-hour urinary NAG, KIM-1, NAG/creatinine, and KIM-1/creatinine levels *(p>0.05)*. There was no significant association between mCl_cr_ levels and duration of treatment or serum drug levels on Spearman’s correlation coefficient *(p>0.05)*.

**Conclusion:**

We found VPA-associated glomerular hyperfiltration in children with epilepsy. We did not observe tubular dysfunction in patients on VPA or LEV.

## INTRODUCTION

Epilepsy is a common neurological condition that affects approximately 0.5% to 1.0% of children and occasionally requires life-long treatment.^1^ Valproic acid (VPA) is commonly prescribed either alone or with other medications for patients with complex partial seizures, as well as simple and complex absence seizures. The administration of VPA has been considered safe but may be associated with adverse effects such as liver and pancreatic damage, brain dysfunction, allergic reactions, weight gain, abnormalities in blood cells, digestive system problems, and dysfunction of the kidney’s glomeruli and tubules.^2^ The normal range for the therapeutic serum levels is typically between 50 and 100 μg/mL. For children, the highest recommended daily dose of VPA is limited to 60 mg/kg.^3^ Levetiracetam (LEV) is a newer drug used as a single medication for patients with partial seizures and in combination with other drugs for generalized seizures. The liver predominantly metabolizes VPA, while LEV has minimal hepatic metabolism and favorable pharmacokinetic properties. LEV generally has minimal side effects and is considered safe, with no known significant drug interactions.^4^ For LEV treatment, the therapeutic range falls between 11 and 32 μg/mL. In pediatric cases, the maximum daily LEV dosage is set at 60 mg/kg.^5^

The exact mechanisms of kidney injury caused by antiepileptic drugs (AEDs) remain unclear. Research conducted on mice has shown that exposure to VPA results in oxidative stress, inflammation, and fibrosis in kidney tissues.^6^ Previous studies have reported that VPA can lead to excessive glomerular filtration, damage to glomerular function, and impairment of renal tubules.^7^ Fanconi syndrome is a potential additional adverse effect that may result from VPA therapy.^2^ However, kidney complications related to LEV are rare and the underlying processes remain poorly understood. Reported adverse effects include interstitial nephritis, acute kidney injury, rhabdomyolysis, hypokalemia, and hypomagnesemia.^8^-^10^

Certain medications can contribute to increased glomerular hyperfiltration and hypertrophy. Adriamycin, puromycin, and streptozotocin can cause pathologies similar to focal segmental glomerulosclerosis (FSGS). Early stages of FSGS-like conditions may show enlarged glomeruli or increased mesangial hypercellularity. Anabolic steroids and interferons can also cause glomerular tuft collapse and extra capillary cell proliferation, with reticular inclusions visible under electron microscopy.^11^

Several biomarkers indicative of kidney damage can be detected in urine or plasma samples. These include cystatin C, neutrophil gelatinase-associated lipocalin (NGAL), kidney injury molecule-1 (KIM-1), various interleukins (IL-6, IL-8, and IL-18), N-acetyl-glucosaminidase (NAG), glutathione transferases (GST), and liver fatty acid binding protein (LFABP). NAG, an enzyme found in the brush border of lysosomes in proximal renal tubular cells, serves as a well-known urinary biomarker indicator of kidney damage. KIM-1 is a tubular-origin transmembrane protein significantly increased in proximal tubule epithelial cells during ischemic or toxic kidney injury in animal models.^12^ 24-hour measured creatinine clearance (mCl_cr_) is widely used to determine the glomerular filtration rate (GFR), a reliable marker of glomerular function.

This study aimed to evaluate renal tubular and glomerular functions using novel biomarkers (NAG and KIM-1) and mCl_cr_ in patients taking VPA and LEV.

## METHODS

The Ankara Training and Research Hospital was the setting for this prospective case-control study between January 2023 and January 2024.

### Ethical Approval:

The research was carried out in compliance with the guidelines specified in the Declaration of Helsinki and received approval from the Ethics Committee of Hospital (approval no: 1166, date: December 28, 2022). Prior to their involvement in the study, all participants provided written informed consent.

Fifty-one epileptic patients participated in this study. Sixteen healthy age and sex matched children without any history of neurological or nephrological diseases were chosen as the control group. The study excluded individuals undergoing treatment with multiple AEDs, as well as those with incomplete data, bedridden patients, concurrent conditions such as hypertension, obesity, or those using steroids or other medications. The patients participated in the study if they had received medication (VPA or LEV) for at least six months. Patient demographics (age and gender), type of epilepsy, duration of illness, duration of treatment, dose, and serum drug levels were extracted from the electronic medical records.

Clinical systolic and diastolic blood pressures were measured using the standard method. A Nihon Kohden bedside monitor (BSM-2301K) with an appropriately sized cuff was utilized to measure office blood pressure. Body mass index (BMI) and z-scores were calculated using reference values for Turkish children.^13^ Blood and urine specimens were collected between 8:00 and 10:00 in the morning for standard analysis, which encompassed blood urea and creatinine levels, fasting blood sugar, aspartate aminotransferase (AST), alanine aminotransferase (ALT), gamma-glutamyl transferase (GGT). Additionally, electrolyte concentrations were assessed, including sodium (Na), potassium (K), calcium (Ca), and phosphorus (P). The analysis also covered uric acid levels and a urinalysis examining pH, density, and specific urine components like Ca, Na, P, creatinine, albumin, and protein.

Patients’ 24-hour urine samples were collected from 8.00 am to 8.00 am the next day. A 10 ml sample was taken from the total volume and placed in a preservative-free tube for laboratory analysis. The Roche autoanalyzer (Roche Diagnostics, Indianapolis, IN, USA) measured total protein, albumin, and creatinine using turbidimetric, immunoturbidimetric, and Jaffe’s kinetic methods, respectively. Post-analysis, samples were stored at -80 °C. Once specimen collection was complete, samples were thawed simultaneously and centrifuged at 3000 rpm for 20 minutes. NAG and KIM parameters were analyzed from the supernatant using the ELISA sandwich principle (SunRed Human ELISA kit).

Creatinine, ALT, AST, GGT, Na, K, and P levels were measured spectrophotometrically using a Roche Cobas analyzer. In whole urine analysis, pH and density were measured by reflectance photometry and refractometry, respectively, with a Roche Cobas analyzer. Spot urine samples were analyzed using the same autoanalyzer for total protein (turbidimetric), albumin (immunoturbidimetric), creatinine (kinetic Jaffe), and calcium (NM-BAPTA method). 24-hour mCl_cr_ was calculated from urinary creatinine excretion and plasma creatinine and indexed for a body surface area of 1.73 m^2^. The tubular phosphorus reabsorption (TPR) and the fractional excretion of sodium (FeNa) were analyzed according to the literature.^14^,^15^

### Statistical Analysis:

Statistical analyses were conducted utilizing SPSS version 21.0 (IBM Corp., Armonk, NY, USA). The Kolmogorov–Smirnov test was employed to evaluate the normality of the data. Descriptive statistics were presented as numbers and percentages for categorical variables and as means and standard deviations for continuous variables. The groups were compared using various statistical tests, including the chi-square test, one-way analysis of variance tests, independent sample t-tests, and post-hoc Tukey tests (in cases where the one-way analysis of variance tests yielded significant results). The correlation between the duration of anti-epileptic treatment, serum anti-epileptic drug dose, and serum GFR levels was determined utilizing the correlation coefficients of Spearman. Statistical significance was found at *p≤ 0.05*.

## RESULTS

This study comprised three groups, the VPA, LEV, and control group, consisting of 30, 21, and 16 patients respectively. Table-I shows the demographic and clinical characteristics of the patients. There were no significant differences between the groups concerning age, gender, or systolic and diastolic blood pressures *(p>0.05)*. However, BMI and BMI z-scores were markedly elevated in the control group compared to the scores of VPA and LEV groups *(p≤0.05)*. There were no significant differences in terms of epilepsy types in VPA and LEV groups. Treatment duration averaged 3.4±1.8 years for the VPA group and 2.8±1.9 years for the LEV group. Serum concentrations were measured at 62.6±26.7 mg/L for VPA and 19.6±17.1 mg/L for LEV. The duration of the illness, the duration of the treatment (year), the dose of the drugs, and serum levels of the drugs are given in Table-II.

**Table-I:** Demographic and clinical characteristics of patients according to groups (VPA, LEV, and control groups).

Characteristics	VPA group (n=30, 44.8%)	LEV group (n=21, 31.3%)	Control group (n=16, 23.9%)	P
Age (years), mean±SD	12.6±3.9	11.0±2.6	12.8±2.6	0.11
*Gender, n (%)* Boys Girls	14 (20.9) 16 (23.9)	8 (11.9) 13 (19.4)	5 (7.4) 11 (16.5)	0.58
BMI (kg/m^2^), mean±SD	18.6±3.9	18.7±4.0	24.3±5.8	0.01*
BMI z-score	0.5±1.5	0.1±1.4	1.1±1.6	0.01*
SBP (mmHg), mean±SD	107.7±15.3	105.8±11.7	113.2±10.8	0.23
DBP (mmHg), mean±SD	64.2±12.3	61.0±9.5	69.7±10.0	0.07
*Type of epilepsy, n (%)* Focal Generalized	3 (5.9) 27 (52.9)	5 (9.8) 16 (31.3)		0.18

* *P* ≤0.05, *Abbreviations:* BMI = body mass index, DBP = diastolic blood pressure, SBP = systolic blood pressure, SD = standard deviation.

**Table-II:** The dosage and the duration of the treatments of VPA and LEV groups.

Characteristics	VPA group (n=30, 44.8%)	LEV group (n=21, 31.3%)
Duration of illness (year), mean±SD	3.4±1.8	2.8±1.9
Duration of treatment (year), mean±SD	3.4±1.8	2.8±1.9
Dose of the drug (mg/kg/day), mean±SD	18.3±5.2	18.6±4.6
Serum level of the drug (mg/L), mean±SD	62.6±26.7	19.6±17.1

*Abbreviations:* SD = standard deviation.

**Fig.1:**
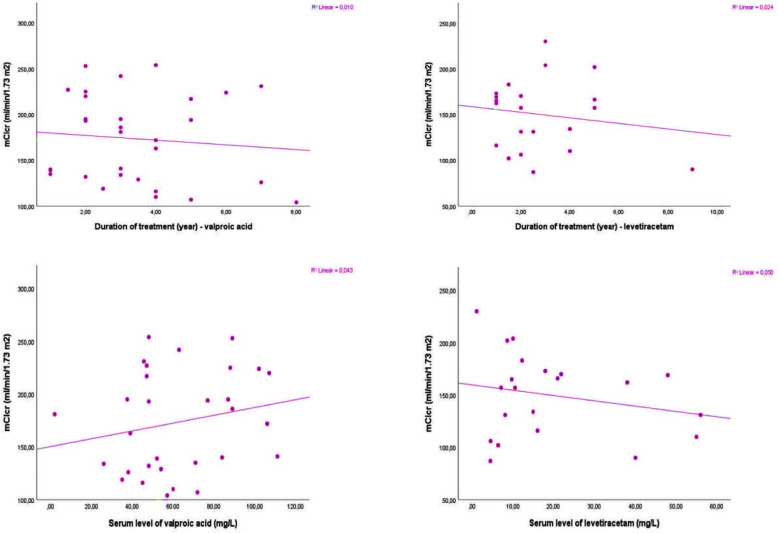
The relationships between mCl_cr_ levels and the duration of treatment and serum levels of drugs in VPA and LEV groups.

In Table-III, comparative serum biochemical levels of VPA, LEV, and control groups are highlighted.. The groups (VPA, LEV, and control group) did not differ in terms of biochemical (Na, K, P, Ca, urea, creatinine, uric acid, AST, ALT, GGT, and glucose levels) parameters *(p>0.05)*. Furthermore, Table-IV shows spot urinary biochemical levels of VPA, LEV, and control groups. In urinary analyses, there was no difference in spot urinary protein/creatinine ratio, spot albumin/creatinine ratio, spot calcium/creatinine ratio, TPR (%), and FeNa (%). Nevertheless, notable variations were observed in urinary pH and mCl_cr_ levels across the groups, with ANOVA results indicating statistical significance at *p=0.02* and *p=0.01*, respectively. The results of the post-hoc Tukey analyses indicated a significant increase in both the mean urinary pH levels and mCl_cr_ levels in the VPA group (6.1±0.7 and 173.4±48.0, respectively) compared to the LEV group (5.6±0.5 and 149.7±39.1, respectively) and control group (5.6±0.6 and 130.6±30.6, respectively). In contrast, the urinary pH and mCl_cr_ levels did not differ between the LEV and control groups *(p>0.05)*. Moreover, there was no significant association between mCl_cr_ levels and duration of treatment (Spearman’s correlation coefficient *p=0.60* and *p=0.50* in VPA and LEV groups, respectively) or serum level of the drug (Spearman’s correlation coefficient *p=0.27* and *p=0.33* in VPA and LEV groups, respectively). The relationships between mCl_cr_ levels and the duration of treatment and serum levels of drugs in VPA and LEV groups are shown in Fig.1.

**Table-III:** The serum biochemical levels of VPA, LEV, and control groups.

Characteristics	VPA group (n=30, 44.8%)	LEV group (n=21, 31.3%)	Control group (n=16, 23.9%)	P
Urea (mg/dL), mean±SD	25.7±6.8	22.3±5.5	22.8±3.9	0.09
Creatinine (mg/dL), mean±SD	0.5±0.1	0.5±0.1	0.6±0.1	0.08
Sodium (mmol/L), mean±SD	139.7±2.1	139.8±1.8	139.7±1.8	0.98
Potassium (mmol/L), mean±SD	4.4±0.4	4.4±0.2	4.5±0.3	0.56
Phosphorus (mg/dL), mean±SD	4.5±0.8	4.8±0.6	4.4±0.5	0.28
Calcium (mg/dL) mean±SD	9.9±0.5	10.1±0.2	9.9±0.4	0.45
Uric acid (mg/dL), mean±SD	4.3±1.2	3.9±1.0	3.9±0.9	0.34
Glucose (mg/dL), mean±SD	92.6±10.2	88.5±22.1	92.7±5.7	0.55
AST (U/L), mean±SD	19.0±8.7	23.7±6.6	21.0±5.9	0.09
ALT (U/L), mean±SD	11.9±3.6	14.9±7.5	15.2±11.0	0.21
GGT (U/L), mean±SD	11.8±5.2	12.9±6.8	12.9±10.4	0.82

*P ≤0.05, *Abbreviations:* ALT=alanine amino transferase, AST=N-acetyl-β-D-glucosaminidase, GGT=gamma-glutamyl transferase, SD=standard deviation.

**Table-IV:** Spot urinary biochemical levels of VPA, LEV, and control groups.

Characteristics	VPA group (n=30, 44.8%)	LEV group (n=21, 31.3%)	Control group (n=16, 23.9%)	P
Urinary pH, mean±SD	6.1±0.7	5.6±0.5	5.6±0.6	0.02*
Urinary density, mean±SD	1022.3±6.3	1022.9±8.4	1021.7±5.9	0.85
Spot urine protein/creatinine ratio (mg/mg), mean±SD	0.26±0.43	0.2±0.14	0.18±0.12	0.60
Spot urine albumin/creatinine ratio (mg/g), mean±SD	57.5±219.4	24.2±41.3	40.3±93.7	0.75
Spot urine calcium/creatinine ratio (mg/mg), mean±SD	0.04±0.06	0.02±0.03	0.06±0.07	0.15
TPR (%), mean±SD	97.6±1.12	97.8±1.16	97.7±0.87	0.89
FeNa (%), mean±SD	0.43±0.28	0.35±0.22	0.38±0.17	0.51

* *P*≤0.05, *Abbreviations:* FeNa=fractional excretion of sodium, SD=standard deviation, TPR=tubular posphorus reabsorption.

Twenty hours urinary biochemical levels and 24 hours urinary NAG shows in Table-V, NAG/creatinine, KIM-1, and KIM-1/creatinine levels of VPA, LEV, and control groups. In 24 hours urinary analyses, there was no difference in 24-hour urinary volume, 24-hour protein excretions, 24-hour creatinine, and 24 hours albumin levels. Furthermore, in terms of 24-hour urinary NAG, NAG/creatinine, KIM-1, and KIM-1/creatinine levels, there were no significant differences between the VPA, LEV, and control groups *(p>0.05)*.

**Table-V:** 24-hour urinary biochemical levels, and 24-hour urinary NAG, NAG/creatinine, KIM-1, and KIM-1/creatinine levels of VPA, LEV, and control groups.

Characteristics	VPA group (n=30, 44.8%)	LEV group (n=21, 31.3%)	Control group (n=16, 23.9%)	P
24-hour urinary volume (ml), mean±SD	1364±989	1247±653	1406±824	0.83
24-hour creatinine (mg/day), mean±SD	931.6±386.2	853.1±478.9	978.5±393.2	0.64
24-hour protein (mg/day), mean±SD	142.8±85.6	136.4±109.5	160.9±111.0	0.75
24-hour albümin (mg/day), mean±SD	22.4±50.9	19.9±47	15.3±31.5	0.88
mCl_cr_ (ml/min/1,73m^2)^, mean±SD	173.4±48.0	149.7±39.1	130.6±30.6	0.01*
24-hour urinary NAG (ng/mL), mean±SD	24.3±5.3	25.7±4.7	23.9±5.1	0.18
24-hour NAG/creatinine (nmol/mg)	0.63±0.54	0.64±0.48	0.52±0.29	0,69
24-hour urinary KIM-1 (ng/mL), mean±SD	4.3±1.0	4.0±1.0	4.6±0.8	0.29
24-hour KIM-1/creatinine (nmol/mg)	0.07±0.05	0.07±0.06	0.06±0.03	0,93

* *P* ≤0.05, *Abbreviations:* KIM-1=kidney injury molecule 1, mCl_cr_=measured creatinine clearance, NAG=N-acetyl-β-D-glucosaminidase, SD=standard deviation.

## DISCUSSION

Our study showed no marked differences between the VPA, LEV, and control groups on NAG, NAG/creatinine, KIM-1, and KIM-1/creatinine levels. In their study, Yüksel et al. documented a rise in NAG activity in 50% of children diagnosed with epilepsy who were prescribed VPA, whereas 17.6% of patients receiving carbamazepine (CBZ) experienced a similar increase in NAG activity during an eight months treatment period.^16^ Similarly, Verotti et al. carried out a research study to evaluate tubular renal function in pediatric and adolescent patients receiving monotherapy with VPA, CBZ, and phenobarbital. Following six months of treatment, individuals treated with VPA and CBZ exhibited a notable rise in the urinary excretion of NAG and β-galactosidase in comparison to their initial and control levels.^17^ According to Altunbasak et al., ambulatory children who took VPA for epilepsy were found to have elevated levels of NAG to creatinine ratios and mean urinary malondialdehyde to creatinine ratios, which may indicate proximal renal tubular dysfunction.^18^ The exact role of AEDs in renal tubular function is not well-established. Evidence suggests that exposing renal tissue to VPA can trigger oxidative stress, inflammation, and fibrosis. Heidari et al. showed that VPA administration to rats resulted in biochemical evidence of Fanconi syndrome. However, they also found that carnitine supplementation improved mitochondrial function and reduced oxidative stress.^2^ In contrast, Hergüner et al. found that female rats administered VPA intraperitoneally for six weeks showed minor reversible biochemical dysfunction and minimal renal fibrosis, as revealed by histopathological studies.^19^ Furthermore, Van Beneden et al. put forward the notion that VPA’s histone deacetylase activity plays a crucial role in safeguarding kidney tissue from inflammation and fibrosis caused by Adriamycin.^20^

In the current study, we observed significantly raised mCl_cr_ levels in the VPA group than in the LEV and control groups suggesting VPA-induced glomerular hyperfiltration. However, the mCl_cr_ levels did not differ between the LEV and control groups. There is no specific cut-off for glomerular hyperfiltration in clinical practice, however, the consensus agreement on a definition of glomerular hyperfiltration based on estimated GFR is >135 ml/min/1.73 m^2^ for children above two years of age.^21^ Similar to our study, Havali et al. found that patients treated with VPA and CBZ showed higher GFR levels, indicating glomerular hyperfiltration.^7^ LEV is a new anti-epileptic drug with favorable pharmacokinetics and low potential for drug interactions.^4^ We did not find evidence of NAG elevation or glomerular hyperfiltration in the patients who received LEV treatment. Therefore, we believe that LEV is a safe and well-tolerated treatment for epilepsy.

The current study did not reveal a substantial correlation between mCl_cr_ levels and the duration of treatment. Unay et al. found a meaningful link between the NAG/creatinine index and treatment period, in children on long-term therapy (>10 years). They also found a significant correlation between the NAG/creatinine index and serum VPA and CBZ concentrations.^22^ Similarly, Hamed et al. revealed a strong association between KIM-1 levels and the duration of treatment with CBZ or VPA.^23^

### Limitation:

It includes the absence of NAG and mCl_cr_ values before treatment. We could not carry out the other markers of kidney injury because we do not routinely use other tubular markers like cystatin C, NGAL, various interleukins (IL-6, IL-8, and IL-18), GST, LFABP, etc. Additionally, the low number of patients and short study period are noteworthy. Further studies with more extended follow-up periods and possibly a diversity in patient populations are necessary to demonstrate the long-term effects of anti-epileptic drugs on renal function.

## CONCLUSION

We found that VPA use in children with epilepsy might be associated with subclinical glomerular dysfunction. However, we did not observe tubular dysfunction as reported by others in patients using VPA or LEV.

### Author Contributions:

**NT, OB, MS, SB, and AY:** Study conception and design. Literature search.

**NT, OB, and AY:** Data collection, analysis, and interpretation Critical analysis.

**NT:** The first draft written of the manuscript. Critical Reiew.

All the authors revised it critically for important intellectual content and approved the final version of the manuscript.

## References

[1] Mehta R, Saini AG, Singhi P, Malhi P (2024). Pattern of Injuries in Children with Epilepsy:A Hospital-Based Case-Control Study. Int J Epilepsy.

[2] Heidari R, Jafari F, Khodaei F, Shirazi Yeganeh B, Niknahad H (2018). Mechanism of valproic acid-induced Fanconi syndrome involves mitochondrial dysfunction and oxidative stress in rat kidney. Nephrology (Carlton).

[3] Young MR, Bisaccia EK, Romantseva L, Hovey SW (2022). Valproic Acid Serum Concentration and Incidence of Toxicity in Pediatric Patients. J Child Neurol.

[4] Sharma D, Hussain AM, Sharma SS (2022). Efficacy of Levetiracetam in neonatal seizures:a systematic review. The Journal of Maternal-Fetal &Neonatal Medicine.

[5] Yamamoto Y, Ohta A, Usui N, Imai K, Kagawa Y, Takahashi Y (2023). Clinical value of therapeutic drug monitoring for levetiracetam in pediatric patients with epilepsy. Brain Dev.

[6] Gezginci-Oktayoglu S, Turkyilmaz IB, Ercin M, Yanardag R, Bolkent S (2016). Vitamin U has a protective effect on valproic acid-induced renal damage due to its anti-oxidant, anti-inflammatory, and anti-fibrotic properties. Protoplasma.

[7] Havali C, Gücüyener K, Buyan N, Yılmaz Ü, Gürkaş E, Gülbahar Ö (2015). Does nephrotoxicity exist in pediatric epileptic patients on valproate or carbamazepine therapy?. J Child Neurol.

[8] Mahta A, Kim RY, Kesari S (2012). Levetiracetam-induced interstitial nephritis in a patient with glioma. J Clin Neurosci.

[9] Thomas L, Mirza MMF, Shaikh NA, Ahmed N (2019). Rhabdomyolysis:a rare adverse effect of levetiracetam. BMJ Case Rep.

[10] Aksoy D, Cevik B, Kurt S, Pekdas E, Solmaz V (2014). Hypokalemia and hypomagnesaemia related to levetiracetam use. J Clin Neurosci.

[11] Radi ZA (2019). Kidney pathophysiology, toxicology, and drug-induced injury in drug development. Int J Toxicol.

[12] Vanmassenhove J, Vanholder R, Nagler E, Van Biesen W (2013). Urinary and serum biomarkers for the diagnosis of acute kidney injury:an in-depth review of the literature. Nephrol Dial Transplant.

[13] Neyzi O, Bundak R, Gökçay G, Günöz H, Furman A, Darendeliler F (2015). Reference Values for Weight, Height, Head Circumference, and Body Mass Index in Turkish Children. J Clin Res Pediatr Endocrinol.

[14] Barth JH, Jones RG, Payne RB (2000). Calculation of renal tubular reabsorption of phosphate:the algorithm performs better than the nomogram. Ann Clin Biochem.

[15] Bhargava S, Jain A, Gupta V (2002). Fractional excretion of sodium--a simple test for the differential diagnosis of acute renal failure. Clin Nephrol.

[16] Yüksel A, Cengiz M, Seven M, Cengiz S, Cenani A (1999). N-acetyl-beta-glucosaminidase and beta-galactosidase activity in children receiving antiepileptic drugs. Pediatr Neurol.

[17] Verrotti A, Greco R, Pascarella R, Matera V, Morgese G, Chiarelli F (2000). Renal tubular function in patients receiving anticonvulsant therapy:a long-term study. Epilepsia.

[18] Altunbaşak S, Yildizaş D, Anarat A, Burgut HR (2001). Renal tubular dysfunction in epileptic children on valproic acid therapy. Pediatr Nephrol.

[19] Hergüner MO, Altunbaşak S, Doğan A, Yildizdaş D, Incecik F, Erdoğan S (2006). Effects of sodium valproate on renal functions in rats. Ren Fail.

[20] Van Beneden K, Geers C, Pauwels M, Mannaerts I, Verbeelen D, van Grunsven LA (2011). Valproic acid attenuates proteinuria and kidney injury. J Am Soc Nephrol.

[21] Pottel H, Adebayo OC, Nkoy AB, Delanaye P (2023). Glomerular hyperfiltration:part 1 - defining the threshold - is the sky the limit?. Pediatr Nephrol.

[22] Unay B, Akin R, Sarici SU, Gok F, Kurt I, Gokcay E (2006). Evaluation of renal tubular function in children taking anti-epileptic treatment. Nephrology (Carlton).

[23] Hamed SA, Rageh TA, Mohamad AO, Abou Elnour SM (2018). Renal dysfunctions/injury in adult epilepsy patients treated with carbamazepine or valproate. Expert Rev Clin Pharmacol.

